# Clinical Relevancy of Circulating Tumor Cells in Breast Cancer: Epithelial or Mesenchymal Characteristics, Single Cells or Clusters?

**DOI:** 10.3390/ijms232012141

**Published:** 2022-10-12

**Authors:** Ivana Fridrichova, Lenka Kalinkova, Sona Ciernikova

**Affiliations:** Department of Genetics, Cancer Research Institute, Biomedical Research Center of Slovak Academy of Sciences, 84505 Bratislava, Slovakia

**Keywords:** breast cancer, circulating tumor cells, clusters, epithelial-to-mesenchymal transition, stemness, clinical utility, evaluation methods, survival, treatment response

## Abstract

Metastatic breast cancer (MBC) is typically an incurable disease with high mortality rates; thus, early identification of metastatic features and disease recurrence through precise biomarkers is crucial. Circulating tumor cells (CTCs) consisting of heterogeneous subpopulations with different morphology and genetic, epigenetic, and gene expression profiles represent promising candidate biomarkers for metastatic potential. The experimentally verified role of epithelial-to-mesenchymal transition in cancer dissemination has not been clearly described in BC patients, but the stemness features of CTCs strongly contributes to metastatic potency. Single CTCs have been shown to be protected in the bloodstream against recognition by the immune system through impaired interactions with T lymphocytes and NK cells, while associations of heterotypic CTC clusters with platelets, leucocytes, neutrophils, tumor-associated macrophages, and fibroblasts improve their tumorigenic behavior. In addition to single CTC and CTC cluster characteristics, we reviewed CTC evaluation methods and clinical studies in early and metastatic BCs. The variable CTC tests were developed based on specific principles and strategies. However, CTC count and the presence of CTC clusters were shown to be most clinically relevant in existing clinical trials. Despite the known progress in CTC research and sampling of BC patients, implementation of CTCs and CTC clusters in routine diagnostic and treatment strategies still requires improvement in detection sensitivity and precise molecular characterizations, focused predominantly on the role of CTC clusters for their higher metastatic potency.

## 1. Introduction

Metastatic breast cancer (MBC) is generally considered an incurable disease; however, new knowledge in the individualized and multidisciplinary approach to curative strategies for selected patients might be a choice for long-lasting remission and improvement of long-term survival in the future [1]. Regardless, the progress evidenced in the past decades in overall survival (OS), 5-year, and 10-year survival rates for breast cancer (BC) patients with primary stage IV are only 27% and 13%, respectively [2].

To date, two main ways for cancer cell dissemination have been identified to occur either through the blood or via lymphatic vessels. Historically, the lymphatic system is considered the primary means for cancer spreading to distant organs. Current experimental observations and clinical trials indicate there is a role of interconnection between the lymphatic and blood circulation that allows metastatic cells to pass from lymphatic nodes to the blood vessels, through the thoracic duct [3]. Cancer cells are then can capable of spreading from the primary tumor site directly by entering the blood vessels or indirectly through the lymphatic system after intensive neo-vascularization or neo-lymphangiogenesis in and around the primary tumor mass [4,5]. In different types of cancers, many factors determine if the spread of cancer is hematogenous or lymphogenous, such as the density of blood and lymphatic vessels, interstitial fluid pressure, tumor hypoxia, and the presence of metastases in regional lymph nodes. In addition, interactions between the tumor tissue and the microenvironment play an indisputable role [5,6].

## 2. Characteristics of Circulating Tumor Cells in Breast Cancer

The lymphatic dissemination of BC documents the intensive lymphovascular invasion inside and around the tumor tissues and metastases in lymph nodes are generally considered the key prognostic markers of cancer spread [4,7]. On the other hand, a growing number of studies have investigated circulating tumor cells (CTCs) showing their role in the hematogenous dissemination of cancer and their clinical utility is under examination in ongoing clinical trials [8]. Among the different cancer types, the CTC presence has been the most widely investigated in BC. Many studies documented that detection and CTC counts were found as independent prognostic factors in patients with both early and advanced MBC [9,10] and CTC appearance predicts the metastatic spread in patients with limited dissemination [11]. Moreover, CTC enumeration became a useful predictor of progression-free (PFS) and OS in MBC patients and could contribute to better management of therapy in individual patients [12,13,14], yet as discussed below, many challenges of CTC clinical utility need to be resolved.

### 2.1. Genetic and Epigenetic Characteristics of CTCs

In peripheral blood of cancer patients, with the exception of apoptotic cancer cells, several other subpopulations of CTCs were found in the form of single cells or organized clusters (discussed later in chapter 3). The introduction of next-generation sequencing and single-cell technologies promotes the characterization of CTC subpopulation heterogeneity. In patients with MBC, homogeneously or heterogeneously mutated *PIK3CA*, *TP53*, and *ESR1* genes and copy number alterations predominantly in *CCND1*, *ERBB2*, and *EGFR* genes were detected in CTC samples [15,16,17,18,19,20,21]. The recent in silico study showed that in patients suffering from several types of cancer, the mutation status between CTCs and primary/metastatic tumors evaluated in multiple genes was extremely different. In BC tissues, the concordance of *PIK3CA* mutations between CTCs and tumor tissues was only 13.73% [22]. Furthermore, notable differences in single-nucleotide variants were found between CTCs and primary BCs [23].

Among the epigenetic changes in CTCs of BC patients, heterogeneous DNA methylation and miRNA expression patterns were detected in several studies. The higher methylation in *CST6*, *BRMS1*, and *SOX17* promoter sequences was observed in patients with operable MBC compared to healthy individuals [24]. Additionally, a significant correlation in *SOX17* methylation between CTC and cell-free DNA (cfDNA) was found predominantly in early BC patients [25]. In another study, at least one of nine genes was methylated in patients’ CTCs compared to samples from healthy donors, and methylation of *CST6*, *ITIH5*, and *RASSF1* genes corresponded with PFS in MBC patients [26]. Furthermore, *ESR1* methylation was detected in both CTC and cfDNA samples from advanced BC patients. In those treated with everolimus/exemestane, the *ESR1* methylation was associated with poor drug response [27]. To investigate the molecular features of CTCs on RNA levels in a high-leukocytes background, the excessive expression of 50 mRNAs and 10 miRNAs was observed in CTC-positive BC patients compared to those in CTC-negative and healthy donors [28]. Other studies have demonstrated a heterogeneous miR-10b expression within CTC subpopulations on a cell-by-cell basis and overexpressed levels of metastasis-associated miR-21 highly correlated between CTCs and corresponding plasma [29,30]. A recent study showed the upregulation of miR-106b expression in CTCs compared to a primary tumor that correlated with epithelial-to-mesenchymal transition (EMT) properties, namely increasing vimentin and decreasing E-cadherin, and poor OS [31].

Many studies have investigated expression profiles of CTCs in patients with MBC and variable concordance and discordance in gene expression profiles between CTCs and primary tumors were identified [32,33,34]. In CTC samples, high levels of *PALB2* and *MYC* genes are associated with a shorter OS and PFS in patients with MBC [35]. Furthermore, the overexpression of *TIMP1* and *AR* genes is associated with worse outcomes and bone metastases, and both genes seem to be the novel therapeutic aims in triple-negative BC (TNBC) [34,36,37].

### 2.2. EMT and Stem-Cell Characteristics in CTCs

In the initial phase of implementation of CTC isolation technologies, the EpCAM-positive CTC fraction was mostly utilized for the investigation of molecular characteristics of CTCs. However, in all BC subtypes, the heterogeneous CTC subpopulations were observed by counting fully epithelial, mesenchymal, and those CTC cells with co-expressing epithelial and mesenchymal markers (E/M hybrids) [38]. Moreover, high-throughput dataset analyses of EMT heterogeneity of tumors and CTCs were performed in several types of cancer including BC. Mathematical modeling predicts different phases of CTCs being equally, less, or more mesenchymal than primary tumors, which are associated with the dynamic of phenotypic transition and cell migration [39]. Cell line experiments showed that highly tumorigenic E/M hybrids were converted to a highly mesenchymal cell population with substantial loss of tumorigenic abilities [40]. E/M hybrid aggressivity could be formed by the combination of both phenotype features, resulting in stem-cell behavior as increased plasticity, self-renewal, mammosphere formation, and production of ALDH1+ cells compared to epithelial cells with less self-renewal and mesenchymal cells with less plasticity [38]. In CTCs of BC patients, E/M hybrids were identified more frequently in MBC patients with poorer PFS and OS [41,42].

The mesenchymal cell phenotype was characterized by increased migratory capacity, invasiveness, and resistance to apoptosis; therefore, EMT has been considered an essential event in BC hematogenous dissemination [43]. Furthermore, TGF-1-induced EMT promotes the chemotaxis-mediated migration of BC cells through the lymphatic vessels [44]. The loss of cell–cell adherent junctions via inhibition of E-cadherin (encoded by the *CDH1* gene) was determined as the key consequence of EMT induction. The most important EMT inducers, which act as direct *CDH1* repressors, belong to three distinct families: the Snail family (SNAIL1, SNAIL2/SLUG, and SNAIL3/SMUC), the Zeb family (ZEB1/2), and b-HLH family (TWIST1/2) [45]. Moreover, *CDH1* expression is inhibited epigenetically by miR-9, miR-23a, and miR-221 direct targeting and in rare cases by promoter hypermethylation [46,47,48,49]. In CTC samples of patients with MBC, the analyses of promoter methylation of three EMT-associated miR-200c/141, miR-200b/a/429, and *CDH1* genes were performed using single-cell multiplexed PCR. The results showed methylation heterogeneity in CTC subpopulations, but contrary to the abovementioned studies, the identified methylation profiles in patients with metastatic tumors were more similar to those of epithelial-like cells [50].

A recent study utilizing the murine and human models of invasive ductal BC showed that the loss of E-cadherin increased cell invasion, but also decreased cancer cell proliferation, survival, CTC number, dissemination in distant organs, and metastasis formation. On the other hand, E-cadherin expression promoted cancer cells at several stages of metastasis in vivo through limiting reactive oxygen-mediated apoptosis [51]. The conception of Ewald and colleagues that loss of E-cadherin is needful for cancer invasion processes and higher E-cadherin expression promotes metastasis [51] was supported by results of another previous study. In this, variable E-cadherin expression was detected, namely strong expression in BCs in situ, moderate in invasive BCs without metastasis, and very weak in tumors with simultaneous lymph node metastasis (LNM) presence, but increasing E-cadherin levels were found in LNM tissues compared to primary tumors [52]. Moreover, this idea corresponds with the fact that higher expression of E-cadherin is associated with shorter survival of invasive BC patients, and its reduced or absent expression is inversely associated with tumor stage in those with ductal BCs [53]. Our recently published results are also consistent with the presented conception. In patients with invasive ductal BCs, decreased levels of E-cadherin encoding gene *CDH1* were found in tumor samples compared to healthy breast tissues, but *CDH1* expression increased in LNMs compared to BC samples [54]. On the other hand, some cell-line results indicated that the EMT process is not dependent on the changes in E-cadherin expression. No relationship was observed in the set of basal BCs undergoing EMT, among which only 50% presented the loss of E-cadherin expression [55].

The mechanisms of EMT are still in debate. Whether it is a strict molecular program initiating the metastatic processes or the result of non-ordered changes in gene expressions resulting in dedifferentiation of epithelial cells to a more primitive state of cells with expression of stem cell markers will require further investigation. In many cancer types, dedifferentiation is frequently found, but it is not clear whether these cells could be precursors for following mesenchymal differentiation [56]. Cell line experiments showed the important role of EMT in cancer cell dissemination, but in human primary tumors, it is not clear, because there are discrepancies in increased expression of mesenchymal markers in the invasive front compared to core cancer tissue [54,56,57].

On the other hand, in transformed human mammary epithelial cells it was found that induction of EMT transcription factors Snail and Twist1 not only led to mesenchymal morphology but also increased expression of CD44^high^/CD24^low^; thereby the stem-cell properties [58]. Furthermore, other cell line study has documented that transition of E/M hybrids to the mesenchymal state is driven by EMT-inducing ZEB1 [40]. More detailed analyses showed the differences in EMT programs between normal mammary stem cells and tumor-initiating cells. In BCs, distinct tumor subpopulations with overexpressed Slug or Snail EMT-transcription factors were found; however, only Slug efficiently promotes the progression of BC cells to the tumor-initiating state [59].

The variable metastatic potential in addition to different morphology, genetic mutations, chromosomal abnormalities, and gene expression profiles was found in highly heterogeneous subpopulations in both primary BCs and patients’ CTC samples [60,61]. However, only cells owning high adaptability and cancer stem cell (CSC)-like features were able to initiate metastasis [62]. Several features have characterized the current CSC model (carefully reviewed by Celià-Terrassa and Jolly [63]). At first, the CSC subpopulation does not need to be minor within the tumor tissue, and its frequency depends on the type and stage of cancer [64]. Secondly, CSCs can be derived not only from normal stem cells but also from any type of adult somatic cells, which have acquired malignant stem-cell features after molecular reprogramming [65]. Thirdly, CSCs form the dynamic population, which obtains or loses stem-cell characteristics named stem cellular plasticity [66]. Finally, CSCs can be highly proliferative, and mostly quiescent and non-quiescent CSCs can coexist similarly to normal stem cells in adult tissues [67].

CSCs from human BCs were firstly isolated in 2003 by Clarke’s team from immunocompromised mice and they were characterized by Lineage^−^/CD24^−/low^/CD44^+^ markers and tumor-initiating capabilities [68]. The predominant stem and progenitor markers CD44^high^/CD24^low^ and ALDH1^high^ were found in both normal mammary epithelial and BC tissues [69,70]. The CD44^high^/CD24^low^ phenotype was associated with primarily quiescent mesenchymal-like cells, which was found at the invasive front of tumors, and proliferative epithelial-like BC stem cells with ALDH1^high^ expression were located more centrally [57]. The role of epithelial-stem features in aggressive BCs was also supported by other studies. In CTC samples, EpCAM^+^ CD44^+^ CD47^+^ MET^+^ and EpCAM^high^/VIM^low^/ALDH1A1^high^ phenotypes were associated with increasing metastatic potency in a xenograft assay and shorter OS and PFS in BC patients [35,71].

**Figure 1:**
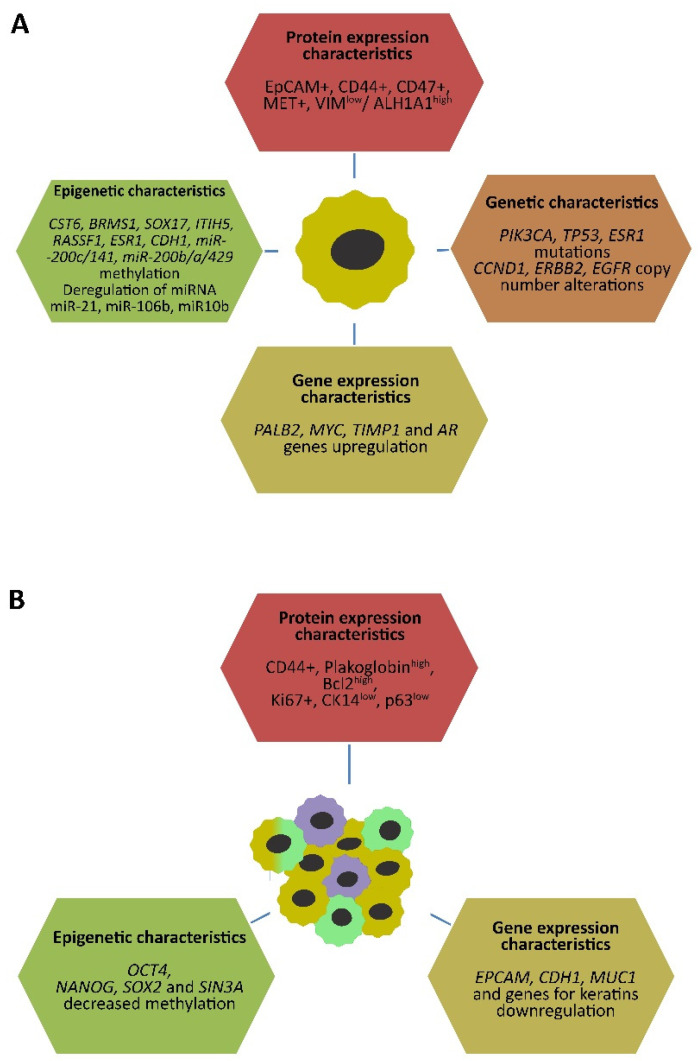
Molecular characteristics for single CTCs (**A**) and CTC clusters (**B**) isolated from breast cancer patients. Described genetic, epigenetic, gene expression, and protein alterations are mostly different from those of primary tumors and heterogeneous within the CTC subpopulations. Abbreviations: CTC, circulating tumor cell (designed by Lenka Kalinkova).

The molecular characteristics of single CTCs have been summarized in Figure 1A.

### 2.3. Interactions of CTCs with Other Cells

The stability of CTCs in blood circulation promotes interactions with other cells and components. The interaction of CTCs with platelets producing TGF-β lead to the likely post-intravasation induction and maintenance of EMT and enhancement of metastasis [72]. In CTC-positive BC patients, a significant increase of CD95 (FAS)+ T-helper cells in peripheral blood samples was found compared to CTC-negative patients [73]. Based on a previously published correlation between CD95 expression and depletion of peripheral blood T-lymphocytes [74], the authors hypothesized that the loss of T-cells in CTC+ patients could result in a loss of long-term antigen activation of CD8 lymphocytes that promote cancer cells’ evasion of the immune attack [73]. Furthermore, CTCs coated with platelets producing MHC-I-positive vesicles sent the “pseudonormal” signal to natural killer (NK) cells, allowing them to escape the NK cell cytotoxicity [75]. These interactions protected CTCs in the bloodstream against recognition by NK and T cells. Furthermore, in BC patients and mouse models, it was documented that the interaction between CTCs and neutrophils stimulates the different gene expression profiles, promoting more intensive metastasis forming [76]. In peripheral blood of cancer patients, including BCs, circulating tumor-associated macrophage-like cells were found binding to CTCs. These giant cells expressed several epithelial, monocytic, and endothelial protein markers. According to the findings, they could cooperate with CTCs in migration processes [77]. In the circulation of solid cancer patients, the cells expressing epithelial and leukocyte markers were found, which originated from the fusion of tumor cells and macrophages. These hybrids with increased growth, motility, invasion capacity, and more active ability of metastasis formation, were also observed in the blood of patients with MBC [78,79].

Intra-tumor heterogeneity corresponds with characteristics of different CTC subpopulations but only partially since the half-life of many of CTCs in the blood of BC patients was observed from 1 to 2.4 h [80]. During the journey in the systemic circulation, CTCs are under constant evolutionary pressure. They are attacked by several events such as immune surveillance, apoptosis, anoikis, and oxidative stress [81,82], leading to the death of most CTCs. Finally, a very small fraction, likely less than 0.01%, is successful in metastasis forming [83,84]. For these reasons, the utility of CTCs as a whole for the evaluation of metastatic potential could be inaccurate. However, the characterization of individual CTC subpopulations could illustrate the clonal evolution during BC progression, and therapy administration in real time. This could be very useful for the personal management of patients [60].

## 3. CTC Clusters in Breast Cancer Dissemination

Although the migrant tumor cells in vasculature named “tumor emboli” (lately termed CTC clusters or circulating tumor microemboli) were firstly described by pathologists in the 19th century, they were poorly investigated. In recent decades, the research endeavor was focused on characteristics of individual CTCs and their clinical utility [85]. CTC clusters were defined as multicellular oligoclonal clumps of 3–100 tumor cells and represented only 2–5% of the total CTC population [86,87]. The half-life of CTC clusters injected in immunodeficient mice was found to be at least three times lower compared to single CTCs, but it was enough time for their seeding into the whole body through the circulatory system [86]. In models of cancer cell invasion into the blood vessels, the grouping of CTC clusters before entering the circulation is considered [81,85,88]. Single CTCs can passively overpass to the blood vessels through the loosely connected endothelial cells, named “leaky” vessels, resulting from VEGF secretion by cancer cells. This occurrence also initiates intensive angiogenesis around the tumor tissue [89]. The intravasation of single CTCs requires reorganization of the cytoskeleton and interactions with the surrounding extracellular matrix, which is promoted by at least the partial EMT phenotype [43]. Small CTC clusters can also utilize this simple mechanism, but the multicellular clusters need active access through the invadopodia and macrophage-dependent manner [90].

Regardless of the idea that CTCs directly detach from the tumor mass in the form of a cluster [86], it was observed in experiments that aggregates may develop from grouped single CTCs inside the blood vessels through intravascular heparanase-mediated cell adhesion [91]. Other authors showed that clusters of tumor cells originated from conjoined individual CTCs rather than collective detachment of cancer cells. Their aggregation was promoted by CD44 homophilic interactions. Moreover, the presence of CTC aggregates with CD44 stem cell features correlated with the poor prognosis of BC patients [92].

In vascular systems, only a small portion of CTCs ultimately develop into macroscopic metastases [84] due to apoptosis, immune attack, and also the squeezing of microcirculation by biomechanical forces. Some CTCs are able to pass through pushed capillaries enabled by adaptive processes such as cellular deformation and nuclear and signaling changes [93]. Multicellular CTC clusters may have a mechanical problem and could be trapped in tiny microvessels. However, the experimental data showed that they were passing through the capillaries ranging from 5 to 10 μm, which was managed by unfolding into single–file chain geometry. In this study, the CTC cluster-mediated dissemination seems to be more effective than cancer seeding performed by single CTCs [94].

### 3.1. CTC Cluster Features

Identification of CTC clusters in the blood of cancer patients has initiated the investigation of their molecular characteristics and metastatic potential. They were detected in 40% of MBC [95], therefore their essential role in advanced cancer has been supposed. The integrity of monoclonal or oligoclonal clumps of CTCs was mediated by cell–cell adhesion proteins including those functioning in tight junctions and desmosomes. Plakoglobin, a desmosomal component, was differently expressed in variable regions within primary BCs, but its upregulation in CTC clusters maintained their integrity [86]. The structure of clusters was also stabilized by the expression of basal epithelial genes as intercellular adhesion molecule cytokeratin-14 (CK-14) and tumor protein P63 contributed to the collective invasion of polyclonal cancer cells. In a mouse model of BC metastasis, it was shown that CTC clusters were stable during the major stages of the metastatic cascade [96].

Similarly, as in single CTCs, the dynamic changes in characteristics and portion of epithelial and mesenchymal components were also found in CTC clusters isolated from BC patients. Moreover, in CTC clusters, E/M hybrid cells were frequently found, which retain epithelial feature of cell–cell adhesion together with mesenchymal characteristics of migration and invasion [97]. An increase in mesenchymal features during the disease progression was observed preferentially in the ductal subtype [61,98]. In lung cancers, CTC clusters expressed more mesenchymal than epithelial markers compared to individual CTCs [99]. However, in TNBC-derived xenografts (TNBC PDX) no differences in EMT-specific gene signature were found between CTC cluster positive vs. negative cases [100], which could indicate the questionable role of mesenchymal phenotype in BC metastasis. In the same study by Trivedi and colleagues, the proteomic and transcriptomic profiles in TNBC PDX models with vs. without CTC clusters were investigated. In the former, the higher expression of Bcl2 protein, apoptosis regulator, and decreased level of acetyl coenzyme A carboxylase-1 (ACC1) was detected. Furthermore, 549 differently expressed genes in association with a CTC cluster presence were identified. Pathway analysis showed the significantly downregulated apoptosis that indicates the important effect of CTC cluster creation on cancer cell maintenance [100]. CTC clusters had a higher percentage of Ki67+ cells compared to matched single CTCs; therefore, they are able actively to proliferate [101]. These authors found the specific DNA hypomethylation patterns in binding sites of transcription factors associated with stemness and proliferation such as *OCT4*, *NANOG*, *SOX2*, and *SIN3A* in CTC clusters compared to single CTCs [101]. The stemness behavior of CTC clusters also contributes to the abovementioned enriched expression of CD44 [92]. The contribution of EMT to the CTC cluster formation was not observed, but higher cell proliferation, stronger stemness features, and lower apoptosis predispose CTC clusters to more active metastatic potential. Conversely, CTC clusters presented low expression of typical CTC markers including *EpCAM*, *CDH1*, *MUC1*, and several genes for keratins [95], which could be useful in their discrimination from single CTCs in blood.

Molecular characteristics of CTC clusters have been depicted in Figure 1B.

### 3.2. CTC Cluster Interactions with Other Cells

In heterotypic CTC clusters, additionally to tumor cells with different proliferating activities, several types of cells modulating the metastatic potential were detected. As was mentioned above, the interaction of single CTCs with platelets was observed in the bloodstream of cancer patients, which induced an EMT-like event [72]. In lung, breast, and melanoma cancer patients, different degrees of platelet coverage of CTC clusters were found. Moreover, these clusters were also captured by leukocytes, indicating that platelets mediated CTC-leukocyte interactions. Such complexes contributed to the creation of early metastatic niche, tumor-vascular interactions, and increasing metastatic growth [102,103,104,105]. However, isolation of such multicomponent clusters for clinical utility requires specific assays, because there are many difficulties with using conventional affinity-based selection methods [102]. Among other blood cells, neutrophils were associated with not only single CTCs, but they also formed CTC-neutrophil clusters, and their adhesion was promoted by neutrophil Mac-1/ICAM-1. This interaction initiated the cell cycle progression and metastatic dissemination [76,106]. Furthermore, tumor-associated macrophages (TAMs) have several functions in metastatic processes. Their essential role is the protection of cancer cells against immune response by producing several cytokines, chemokines, and growth factors [88,107,108]. On the other hand, after the integration with single CTCs or within CTC clusters, TAMs can promote cancer cell intravasation, extravasation, vascularization, EMT initiation, and forming of pre-metastatic niches [109,110,111,112,113]. In BCs, two TAM subpopulations with different molecular profiles and specialized functions were identified according to the location within the tumor and interactions with mediators secreted by surrounding cells. In the perivascular area, the pro-metastatic migratory TAMs characterized as M1-like were located. Inversely, pro-angiogenic sessile TAMs that resembled M2-like phenotype were found in tumor-stroma borders and/or hypoxic regions [107]. Generally, the abovementioned blood cells as the components of CTC clusters improve their tumorigenic features; however, the impaired interactions of T cells and NK cells with single CTCs protected them from immune evasion.

Other associations supporting the cancer progression were observed between CTCs and stroma cells. The previously published model of metastasis presented the role of tumor cell attachment to endothelium in metastasis initiation. In a mouse model, lung tumor cells were connected to endothelia of pulmonary arterioles or capillaries, and only these attached cells presented the source for secondary tumors [114]. However, in association with BCs, this feature has not been investigated yet. Other cells promoting BC initiation, progression, and metastasis are cancer-associated fibroblasts (CAFs) [115]. In addition, they contribute to drug resistance, immune evasion, and stemness in BC [116,117,118].

A previous study showed that metastatic cells could bring the stromal components, including CAFs, from the primary tumor to metastasis. The brain metastasis originating from lung, renal cell, and breast carcinomas incorporated CAFs when compared to primary brain tumors or healthy brain tissues. The authors also observed that metastatic CTCs from blood samples were more viable when integrated into heterotypic clusters consisting of tumor and stromal cells [119]. Furthermore, significantly higher numbers of both CTCs and circulating CAFs were found in the peripheral blood of BC patients with metastatic disease compared to patients with localized BC [120]. A recent report documented that CTCs, CAFs, and clusters were seeded in the early stage of BC and they were observed during all stages of disease progression. Experiments in vivo showed the higher metastatic potential of CTC–CAF heterotypic clusters compared to homotypic CTC clusters. Moreover, the authors documented the involvement of adhesion and the stemness CD44 marker in such CTC–CAF heterotypic clustering [121]. The ability of the clustering function of the CD44 marker was previously discussed in relation to homophilic CTC aggregation [92].

**Figure 2:**
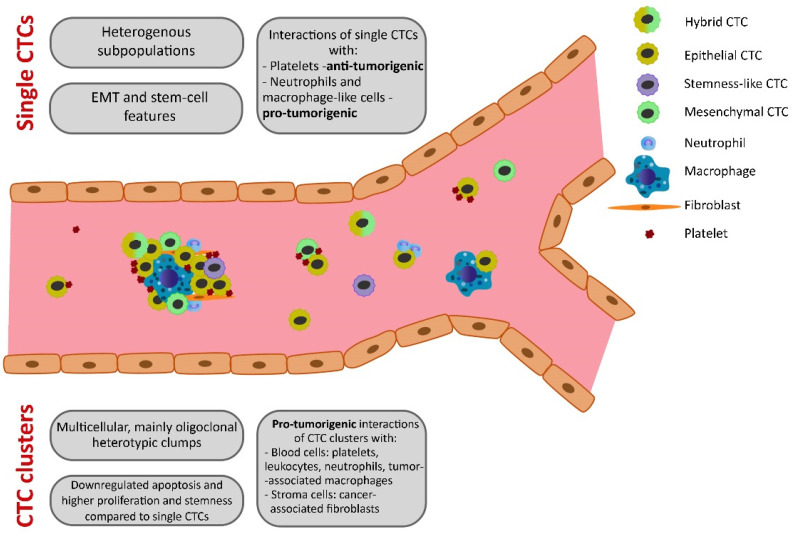
Tumorigenic characteristics and interactions of disseminated breast cancer cells. Presented features of CTC clusters and their pro-tumorigenic interactions with several types of cells indicate higher metastatic potency compared to single CTCs. Abbreviations: CTC, circulating tumor cell; EMT, epithelial-to-mesenchymal transition (designed by Lenka Kalinkova).

Tumorigenic characteristics and interactions of disseminated breast cancer cells with other cells have been summarized for CTCs and CTC clusters in Figure 2.

Presented results documented that clustering of CTCs in both homotypic and heterotypic forms, joined with several types of blood and stroma cells, significantly increased the metastatic potential of BCs. Therefore, the detailed investigation of molecular features and behavior of CTC clusters, with emphasis on standardization of CTC cluster isolation methods, brings more precise tools for the prognosis of metastatic potential and recurrence monitoring in BC patients.

## 4. Clinical Utility of CTCs

### 4.1. Methods of CTC and CTC Cluster Evaluations

The low number of CTCs in circulation, compared to hematopoietic cells and other blood compartments, together with their ability to form aggregates (CTC clusters) highlight the need to create specific techniques for their enumeration, isolation, detection, and clear phenotypization. To date, many technologies have been developed targeting CTCs based on CTCs´ physical and biological properties (reviewed in [8,122,123,124]).

CTC enrichment methods based on biological properties applied a positive or negative selection of CTCs from other blood cells, respectively. These approaches use immunoaffinity assays which separate CTCs and non-malignant cells based on expression-specific surface proteins [124].

For instance, the only FDA-approved CellSearch^®^ System (Menarini Silicon Biosystems, Florence, Italy) is based on the selection of EpCAM-positive CTCs using an immunomagnetic device and is useful for diagnostic, prognostic, and predictive purposes in BC patients [125,126]. Similarly, other positive enrichment immunomagnetic methods are used in BC CTC analysis, such as MagSweeper^TM^ (Illumina, San Diego, CA, USA) or AdnaTest^®^ Breast Cancer system (Qiagen, Hilden, Germany). Using MagSweeper separation from the BC patients´ tissues and blood, living single cells were screened for mutations in exons 9 and 20 of the *PIK3CA* gene [16]. AdnaTest^®^ presents a magnetic beads-based CTC detection method with subsequent RT-PCR characterization of epithelial–mesenchymal markers such as EpCAM, MUC1, EGFR, and HER2, and in addition to isolation could complement the CellSearch^®^ system by genomic marker status in CTCs [127,128]. Positive selection based on magnetic-activated cell sorting (MACS) using magnetic beads conjugated with EpCAM was successful in BC CTCs’ enrichment. However, in the clinical study, intact morphology of tumor cells was not detected. This suggests that MACS with a standard cytological routine is not suitable for common CTC diagnostics in early BC patients [129].

Another group of positive selection CTC methods includes microfluidic chips with specific antibodies which efficiently bind to CTC antigens. Microfluidic devices divide into micro-post (CTC chip) [130] and surface-based methods, such as graphene oxide (GO) chip, Herringbone chip (HB Chip) [131,132], and GEDI chip. These use the principle of geometrically enhanced differential immunocapture with specific HER2 antigen applicable to CTC detection of HER2-expressing BC [133]. Similarly, modified versions of microfluidic devices improve CTC capture and CTC recovery; for example, Park et al. invented gold nanoparticles bound to an HB chip to allow capture of CTCs, and subsequent exchange for chemical ligand resulted in cell release. This technique possesses several advantages compared to classical HB chips [134].

Unfortunately, the mentioned in vitro methods have blood volume limitations, but the in vivo CellCollector^®^ (Gilupi, Potsdam, Germany) method allows CTC isolation and identification directly from BC patients’ circulation. Tumor cells were characterized as CD45-negative, nuclear-positive, and CK-positive, while CD45-positive, nuclear-positive, and CK-negative cells were identified as leukocytes. BC patients with ≥3 CTC had shorter progression-free survival (PFS) and overall survival (OS), which suggested that the CellCollector^®^ method has prognostic potential in BC [135].

Positive selection methods that are mostly based on EpCAM antigen-antibody bound, leading to the loss of the EMT subpopulation of CTCs. Contrasting procedures applied negative selection to remove peripheral mononuclear cells using specific markers on blood cell surfaces such as CD66b or CD45 [124]. Typically, the EasySep^TM^ (StemCell Technologies, Vancouver, Canada) and DynaBeads^®^ (Thermo Fisher Scientific, Waltham, MA, USA) CD45 systems of antigen depletion based on immunomagnetic procedures are used for CTC enrichment of BC patients [136,137]. Similarly, the CD45 selection method RossetteSep^TM^ system (StemCell Technologies, Vancouver, BC, Canada) depletes CD45^+^ blood cells with a second step, based on density centrifugation, and obtains a cell population enriched by CTCs characterized by epithelial and mesenchymal markers analyzed by qRT-PCR. The population of tumor cells isolated by this technique includes subpopulations of epithelial and mesenchymal CTCs which are associated with BC patients´ outcomes and clinical characteristics [138,139].

CTC-iChip represents an immunomagnetic sorting method, which can have three modes: label-free, and positive or negative selection according to used antigens, EpCAM for epithelial CTCs and CD45, and CD15 marker for leukocytes and granulocytes, respectively [140].

The EPISPOT functional assay, detecting exclusively living CTCs and disseminated tumor cells (DTC), is based on the detection of proteins secreted by CTCs combined with leukocyte depletion. The analysis which combines both CellSearch^®^ and EPISPOT methods has determined the best predictor of OS in MBC patients followed by EPISPOT and CellSearch^®^ analysis alone [141].

The next subcategory of CTC technologies using the separation and detection of physical properties characteristic of tumor or blood cells are based on a label-independent method, and therein may provide an advantage in the form of subsequent molecular analyses of CTCs. Tumor cells differ by cell surface charge (negative) and also cytoplasmatic conductivity from white blood cells [124]. The ApoStream^®^ (ApoCell, Houston, TX, USA) and DEPArray^TM^ (Menarini Silicon Biosystems, Florence, Italy) platforms use a marker selection-free approach based on dielectrophoresis to separate and capture CTCs or CTC clusters, respectively [142,143]. ApoStream^®^, in combination with additional CTC characterization, was proven to separate 3 CTC phenotypes (epithelial CTCs, EMT-CTCs, cancer stem-like cells CTCs) in BC patients who received neoadjuvant chemotherapy and correlated them to overall pathological response [142].

Gradient technologies such as Accucyte^®^-CyteFinder^®^ (RareCyte, Seattle, WA, USA) and OncoQuick^®^ (Greiner Bio-One, Frickenhausen, Germany) present sensitive and comprehensive systems based on cell density. This allows the separation and further characterization of CTCs including whole-genome amplification or proliferative activity analysis for a more detailed characterization of CTCs’ chemoresistance in BC patients [144,145,146].

Since tumor cells can have a larger diameter and morphology than blood cells, several methodologies were developed based on filtration according to size and/or deformability. Moreover, many of these methods are useful for CTC cluster separation and identification. Commercially available size-based technologies include ISET^®^ (RareCells Diagnostics, Paris, France), ScreenCell^®^ (ScreenCell, Sarcelles, France), Parsortix^®^ (Angle plc, Surrey, UK), CellSieve^TM^ (Creatv MicroTech, Potomac, MD, USA), and ClearCell^®^ FX (Clearbridge Biomedics, Singapore), with sensitive and specific CTC separation from whole blood of BC patients [137,147,148,149,150]. Another promising filtration system, a flexible micro spring array (FMSA) device, can enrich viable CTCs with 90% capture efficiency directly from whole blood. This method detected at least one CTC in approximately 76% compared to 22% of clinical samples analyzed by the CellSearch^®^ system [151]. The ClearCell^®^ FX system utilizes the dean flow fractionation (DFF) principle in a spiral microfluidic device and allows high specificity and sensitivity with intact cell retrieval [152]. In addition, this method proved the prognostic potential of counting CTCs before treatment as an independent predictor of PFS in MBC patients [150].

Likewise, many other non-commercial methods were developed using sized-based microfluidic devices able to isolate single CTCs or clusters in testing of BC patient samples [95,153,154,155,156,157,158]. Similarly, the Vortex chip uses micro-scale vortices and inertial focusing to selectively isolate and concentrate larger CTCs [159].

Currently, advancing nanotechnology methods, including various types of nanomaterials and nanostructured substrates, are being developed for CTC detection. These include gold nanoparticles, magnetic nanoparticles, graphene, carbon nanotubes, quantum dots, and dendrimers [160]. The nanotube-CTC chip is a new 76-element microarray technology that combines carbon nanotube surfaces with microarray manufacturing techniques based on the physical mechanisms of preferential adherence of CTCs on a nanotube surface similar to collagen adhesion matrix (CAM) scaffolding, reported as a viable approach for CTC capture in patients. This chip successfully captured CTCs in the peripheral blood of BC patients (stage 1–4) with a range of 4–238 CTCs per 8.5 mL of blood and with >90% cancer cell adherence compared to 50% to CAM [161].

Lastly, a separate group of direct imaging methods, in vitro and/or in vivo, are presented. Mostly, these scanning and imaging techniques are based on flow cytometry. For instance, CytoTrack^TM^ (CytoTrack, Lyngby, Denmark) was used to map individual CTCs [162] or FAST (fiber-optic array scanning technology) based on digital capturing of labeled cells’ fluorescence stimulated by laser, enabling rapid location of CTCs [163]. A very promising new diagnostic method, able to reveal and identify CTCs and CTC clusters, is photodiagnostic infrared spectroscopy (PDIS). In addition to the detection of the presence or absence of CTCs and clusters in blood circulation with 98% sensitivity, it distinguishes various CTC cluster phenotypes [164].

Despite the development of many CTCs technologies to date, improving and designing new methods, combined with new knowledge about molecular characteristics of both individual and CTC clusters, have great potential for utilizing CTC methods and analyses in BC clinical practice. Here we have reported and summarized CTC evaluation methods used in BC patients´ blood samples (Table 1).

### 4.2. Clinical Utility of CTC and CTC Cluster Analyses in Breast Cancer

**Figure 3:**
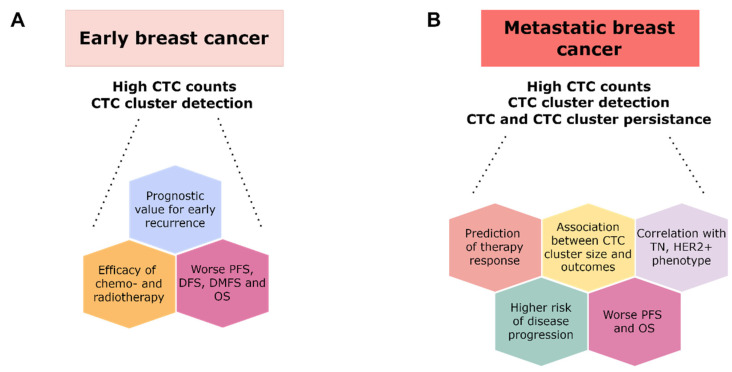
Clinical relevance of CTC and CTC cluster count and persistence evaluation utilized in patients with early (**A**) and metastatic breast cancer (MBC) (**B**). Mounting evidence regarding the clinical relevance of CTCs and CTC clusters comes from studies comprising MBC. Since the improvements in detection methods, an emerging role of CTCs and CTC clusters was documented in early BC, showing the correlations with early recurrence, treatment response, and patient outcomes. However, many associations are not clearly defined, and further research in large cohorts of early BC, as well as MBC, patients might help to clarify the real relationships. Abbreviations: CTC, circulating tumor cell; DFS, disease-free survival; DMFS, distant-metastasis-free survival; HER2+, human epidermal growth factor receptor 2 overexpressed; MBC, metastatic breast cancer; OS, overall survival; PFS, progression-free survival; TN, triple negative.

The clinical relevance and prognostic value of CTCs and CTC clusters were validated in both early and metastatic BC (Figure 3). Most studies have reported on epithelial-enriched clusters using the CellSearch^®^ system since it is the only FDA-approved platform suitable for CTC quantification in clinics. Targeting the CTCs may serve as an early predictive marker of poor PFS and OS, monitoring the benefit of anti-cancer treatment. Despite the proven association between a high CTC count and poor prognosis of various BC stages, the implementation of CTCs and CTC clusters in routine diagnostic and treatment strategies still needs to be determined and requires further studies.

#### 4.2.1. Studies on Early Breast Cancer

CTC and CTC cluster occurrence in patients with early BC has not been studied in such detail as metastatic malignancies. A correlation between CTCs and tumor size, grade, hormone receptors (HR), HER2, and axillary lymph node status was evaluated in 302 chemo naive and non-MBC patients. At least one CTC identified by CellSearch^®^ System was observed in 24% of patients. This clinical study suggested an important prognostic value of CTC detection information in early recurrence and patient outcomes, showing a statistically significant decrease in both PFS and OS ([HR] 4.62, 95% CI 1.79–11.9 and HR 4.04, 1.28–12.8, respectively) in CTC-positive patients [166]. The presence of CTCs was shown to be an independent prognostic marker also in adjuvant chemotherapy settings. In this context, the presence of CTCs was monitored in two large cohorts containing 2026 early BC patients before chemotherapy and 1492 after the treatment. According to the results, 21.5% of patients before adjuvant chemotherapy were CTCs-positive. Importantly, CTCs were detected in 22.1% of patients after chemotherapy and their presence was significantly associated with poor disease-free survival (DFS) as well as poor OS. However, no correlation was found with tumor size, grading, or HR status [167].

Conversely, a pooled analysis of individual data from 3173 non-metastatic BC patients showed that the presence of CTCs correlated with higher tumor size and lymph node involvement. Importantly, CTC counts served as an independent prognostic factor for disease-free (DF) as well as OS (HR = 1.8; 95% CI 1.5–2.3 and HR = 2; 95% CI, 1.5–2.6, respectively) [168]. Accordingly, the results of an international meta-analysis containing more than 2000 non-metastatic BC patients on neoadjuvant chemotherapy (IMENEO study) showed a strong association between CTC counts and distant-metastasis-free survival, OS, and locoregional relapses [169]. Importantly, the statistical significance positively correlated with increasing CTC counts [169].

A beneficial effect of adjuvant radiotherapy on relapse-free survival and/or OS has been documented in CTC-positive early-stage BC patients included in the National Cancer Database (NCDB) and in a multicenter phase 3 SUCCESS clinical trial (Simultaneous Study of Gemcitabine–Docetaxel Combination Adjuvant Treatment as well as Extended Bisphosphonate and Surveillance). CTCs were detected in 23.5% out of 1697 patients in NCDB and 19.4% out of 1516 patients enrolled in the SUCCESS cohort. Multivariable analyses showed longer OS in CTC-positive patients receiving radiotherapy compared to those who did not undergo this treatment modality [170].

The results of a large randomized phase II trial EORTC 90091-10093 BIG 1–12 Treat CTC comprising patients from 70 hospitals across 5 European countries reported no decrease in the CTC detection rate in HER2 nonamplified, early BC after treatment with trastuzumab [171]. Recently, Reduzzi et al. have documented a higher CTC cluster detection rate in 19 clinical samples processed with CellSieve^TM^ filters compare to CellSearch^®^ detection. Interestingly, the processing of samples with marker-independent ScreenCell^®^ filters found more CTC clusters in early BC compared to metastatic disease, showing CTC cluster formation and dissemination are early events in breast carcinogenesis [145]. This is in line with the evidence proving the presence of CTC clusters in early BC with the establishment of the Smart BioSurface slides (SBS-CTC) technology in combination with the CellSeed device [172].

#### 4.2.2. Studies on Metastatic Breast Cancer

Since there is a proven role of CTCs and CTC clusters in tumor spreading, most studies concerning the clinical relevance have been limited to patients with metastatic or advanced disease. Cristofanilli et al. firstly reported the clinical validity of CTC count by the CellSearch^®^ system) on 177 BC patients. The results showed that CTCs were detected in approximately 60% of patients with significantly worse PFS and OS in those with a CTC count of ≥5 cells per 7.5 mL of blood [12]. The important role of CTC enumeration for disease stratification was also confirmed in a large retrospective international analysis with significantly shorter OS in patients stratified as stage IV aggressive BC patients with ≥5 cells per 7.5 mL of blood compared to stage IV indolent (<5 cells per 7.5 mL of blood) (15.4 months vs. 37.1 months, respectively) [173].

Paoletti et al. observed that approximately one-third of triple-negative MBC (TN MBC) patients enrolled in a prospective phase II trial of nanoparticle albumin-bound paclitaxel (nab-paclitaxel), with or without tigatuzumabs, was CTC-positive at baseline and days 15 and 29. Moreover, a significant impact of residual cluster occurrence on these days on PFS was shown. However, no significant differences in PFS have been observed between patients with CTC clusters and those without at the baseline [174]. A randomized phase III SWOG S0500, determining the CTC levels in 595 MBC patients before and during first-line chemotherapy, recorded increased CTC counts in 123 patients before and after the first cycle of treatment. According to study results, CTCs have a strong prognostic significance in MBC patients receiving first-line chemotherapy. However, changes in the chemotherapy regime based on CTC persistence did not correlate with increased PFS or OS, suggesting that persistent CTCs ≥ 5 during systemic treatment may be coupled with chemotherapy resistance [175]. A meta-analysis pooling 50 studies with 6712 BC patients analyzed the changes in CTC status prior to and after different anticancer treatment modalities, demonstrating that CTC status could predict the treatment response in patients with BC in metastatic settings [176]. However, a recent multicenter randomized clinical trial CirCe01, conducted to monitor the CTC counts in MBC patients after two lines of chemotherapy, failed to demonstrate the clinical utility of CTC monitoring in MBC patients after the third line of chemotherapy due to the limited accrual and compliance [177].

A retrospective translational medicine study was designed to evaluate the impact of CTC doublets or clusters on the prognosis of MBC patients who participated in the SWOG S0500 clinical trial. Survival analysis methods, including Kaplan–Meier plots and log-rank tests, were used to re-read data achieved in SWOG S0500. This showed no prognostic value of CTC doublets or clusters in MBC patients with elevated CTC counts (5–19 CTCs or ≥50 CTCs per 7.5 mL of blood). These findings suggested that mortality is associated with the number of CTCs rather than with the presence of CTC clusters [178]. On the other hand, longitudinal enumeration and CTC cluster evaluation performed on 156 women with newly diagnosed MBC reported significantly worse survival in patients with CTC clusters compared to those without clusters. The results confirmed a correlation between changes in CTC counts during the treatment and survival, pointing out the prognostic value and clinical relevance of CTC evaluation. As shown, patients with persistent CTCs ≥5 were characterized by worse PFS and OS compared to those with elevated CTCs at baseline (≥5 CTCs per 7.5 mL of blood) but decreased CTC counts in follow-up samples [179]. In a currently published study, Costa et al. used the CellSearch^®^ system to isolate and evaluate the prognostic value of CTCs and CTC clusters in longitudinally collected blood samples from 54 MBC patients. According to the results, elevated CTC counts and CTC clusters at baseline were significantly associated with a higher risk of disease progression and significantly shorter survival. In addition, the results showed a positive relationship between CTC cluster size and patient outcome [180].

A prospective study comprising 115 advanced-stage BC patients before anticancer treatment, and during the follow-up period, investigated the prognostic significance of CTC clusters compared to single CTC counts. They found that elevated levels of single CTCs (≥5 cells per 7.5 mL of blood), and the presence of CTC clusters at baseline (detected in 31.3% and 17.4% of patients, respectively), were associated with significantly worse PFS. In addition, patients with both elevated CTCs and clusters had an increased risk of disease progression, suggesting that CTC clusters have an additional prognostic value in the outcomes of BC patients [181]. The analysis of longitudinally collected CTCs and CTC clusters in 128 MBC patients during a 2-year follow-up confirmed the significant associations between CTCs and CTC clusters with patient PFS and OS using Cox proportional hazards models. The results showed a hazard ratio (HR) of 7.96 (95% confidence level (CI) 2.00–31.61, *p* = 0.003) and 14.50 (3.98–52.80, *p* = 0.001) for the presence of 2-cell or 3-cell CTC clusters, respectively. Importantly, larger CTC clusters were linked to a higher risk of death in MBC patients [182].

Regarding the association between CTCs and HR status in MBC, a prospective observational cohort of 52 MBC patients showed that the presence of CTC clusters in blood was more common in patients with TN and HER2-positive BC than in patients with HR-positive malignities. Interestingly, morphologic features of CTCs and CTC clusters were not associated with prognosis at baseline. However, detection of apoptotic CTCs or clusters in follow-up blood samples during systemic therapy correlated with a worse prognosis in terms of PFS and OS [183].

In summary, mounting evidence from clinical trials comprising large cohorts of BC patients documented a significant prognostic value of CTC and CTC cluster detection in early as well as metastatic disease [184]. Studies confirmed the clinical relevance of CTC enumeration and the presence of CTC clusters and their relationship to patient outcomes. Importantly, ongoing clinical trials concerning the role of CTC in BC treatment are listed in Table S1. Although morphological and biological features of CTCs might play a role in metastatic dissemination, comprehensive studies concerning this issue are still missing.

## 5. Conclusions

In the era of precision medicine, the implementation of non-invasive tumor screening methods in the standard diagnostic and treatment protocols represents a huge challenge for clinical oncology. Importantly, CTC detection plays a critical role in diagnosis, early detection, and evaluation of chemotherapy efficacy and malignancy recurrence. Since there is a strong metastatic potential of BC and far better prognosis for low-grade breast tumors, early-stage detection of CTCs is crucial in terms of clinical outcomes for patients. Compared to conventional imaging or tissue biopsy, CTC detection and other molecular characterization of CTC subpopulations may serve for real-time monitoring of disease progression. Moreover, CTC-driven procedures may identify the differences in the mutation status and epigenetic alterations in the expression of cancer-related genes between solid tumors and CTCs. Several studies reported that changes in CTC counts during cancer therapy could predict treatment efficacy. Many studies have investigated the particular genetic, epigenetic, epithelial, or mesenchymal-like features of CTCs, as well as identifying the specific cell-to-cell interactions within mixed CTC clusters. The synthesis of present knowledge, other in silico analyses, and the development of machine-learning algorithms would be of great interest to targeting the clinically most relevant CTC population. Importantly, the relationship between CTCs and both the tumor microenvironment and the immune system is still poorly understood and needs to be investigated.

In conclusion, the potential for routine application of CTC detection in oncology is enormous. Mounting evidence highlights the metastatic potential of CTCs in many cancer types, including BC. However, several concerns need to be addressed, including a rare concentration of CTC circulating in the bloodstream and the determination of specific characteristics of the CTC subtypes. Moreover, a comprehensive approach should resolve the technical issues coupled with sample extraction procedures and overcome the limitations of methods of CTC cluster evaluation in terms of disruptive elements that could damage multicellular aggregates. The only FDA-approved system for CTC isolation opens up the possibilities for researchers to improve other platforms aiming to increase CTC and CTC cluster detection. In this context, a combination of methods considering both biological and physical properties of CTCs seems to be a perspective trend. Further research and clinical studies are warranted to increase the sensitivity of CTC detection methods and establish standardized protocols and standards for use in the clinical setting. Significantly, a more detailed study of CTC cluster formation and dissemination might bring new insights into the mechanism of metastasis, leading to the development of new diagnostic and anti-cluster therapeutic strategies for early BC manifestation.

## Figures and Tables

**Table 1:** Single CTCs and/or CTC clusters detection technologies in breast cancer clinical samples.

Technique	Selection	Form of Disseminated Cancer Cells	References
*Technologies based on biological properties*			
CellSearch^®^	EpCAM-positive selection	CTCs, CTC clusters	[126,149]
MagSweeper^TM^	EpCAM-positive selection	CTCs	[16]
AdnaTest^®^	EpCAM-positive selection + RT-PCR	CTCs	[127,128]
MACS	EpCAM-positive selection	CTCs	[129]
CTC chip	EpCAM-positive selection	CTCs	[130]
GO chip	EpCAM-positive selection	CTCs	[131]
HB chip	EpCAM-positive selection	CTCs, CTC clusters	[132,134]
GEDI chip	HER2- positive selection	CTCs	[133]
CellCollector^®^	EpCAM-positive selection	CTCs, CTC clusters	[135]
EasySep^TM^	Negative selection (CD2, CD14, CD16, CD19, CD45, CD61, CD66b, and Glycophorin A depletion)	CTCs	[136,137]
DynaBeads^®^	Negative selection (CD45 depletion)	CTCs	[137]
RossetteSep^TM^	Negative selection (CD45 depletion) and density gradient centrifugation	CTCs	[138,139]
CTC-iChip	Positive or negative selection, antigen-independent	CTCs	[140]
EPISPOT	Negative selection with protein secretion	CTCs	[141]
*Technologies based on physical properties*			
ApoStream^®^	Dielectrophoresis	CTCs	[142]
DEPArray^TM^	Dielectrophoresis	CTCs, CTC clusters	[143]
Accucyte^®^-CyteFinder^®^	Cell density (Accucyte) with subsequent immunofluorescence staining (CyteFinder)	CTCs	[144,145]
OncoQuick^®^	Cell density	CTCs	[146]
ISET^®^	Size	CTCs	[147]
ScreenCell^®^	Size	CTCs, CTC cluster	[137,149]
Parsortix^®^	Size and deformability	CTCs, CTC clusters	[148,165]
CellSieve^TM^	Size	CTCs, CTC clusters	[149]
ClearCell^®^ FX	Size, inertial focusing	CTCs	[150]
FMSA	Size and deformability	CTCs, CTC microclusters	[151]
Vortex chip	Size, inertial focusing	CTCs, CTC clusters	[159]
p-MOFF device	Size, inertial focusing	CTCs	[153]
Cascaded spiral microfluidic device	Size, inertial focusing	CTCs	[154]
Cluster-chip	Size, cell–cell junctions	CTC clusters	[95]
DLD chip	Size and asymmetry	CTC clusters	[155]
Micro-ellipse filters	Size and deformability	CTCs	[156]
Microscope-slide-sized PDMS	Size	CTC clusters	[157]
Hexagonal microfluidic chip	Size	CTCs, CTC clusters	[158]
Nanotube CTC chip	Preferential adherence	CTCs	[161]
*Direct imaging technologies*			
CytoTrack^TM^	Flow cytometry and fluorescence microscopy with previous cell density enrichment	CTCs	[162]
FAST	Laser-scanning	CTCs	[163]
PDIS	Photodiagnostic and spectroscopy	CTCs, CTC clusters	[164]

Abbreviations: DLD, deterministic lateral displacement; FAST, fiber-optic array scanning; FMSA, flexible micro spring array; GEDI, geometrically enhanced differential immunocapture technology; GO, graphene oxide; HB, herringbone; MACS-, magnetic-activated cell sorting; MOFF, multi-orifice flow fractionation; PDIS, photodiagnostic infrared spectroscopy; PDMS, polydimethylsiloxane.

## Data Availability

Not applicable.

## Supplementary Materials

The following supporting information can be downloaded at: https://www.mdpi.com/article/10.3390/ijms232012141/s1.
