# Supplementary material for: Clinical Relevancy of Circulating Tumor Cells in Breast Cancer: Epithelial or Mesenchymal Characteristics, Single Cells or Clusters?
Source: Int J Mol Sci. 2022 Oct 12;23(20):12141. doi: 10.3390/ijms232012141 (PMC9603393; doi:10.3390/ijms232012141)
Supplement: Supplementary file 1 [file ijms-23-12141-s001.zip › ijms-1940973-supplementary.pdf]

Table S1. Evaluating the role of circulating tumor cells in breast cancer treatment. The table summarizes the list of ongoing clinical trials concerning the relationship between circulating tumor cells (CTCs) and early and metastatic breast cancer (BC) treatment (according to https://clinicaltrials.gov/).

Identifier	Study design	Purpose	Patients (n)	Intervention/treatment	Study status
**NCT02965755**	An interventional, a single-arm, open-label study	To determine whether data from patients´ blood and tumor tissue are comparable in determining research-based recommendations for cancer treatment. Changes in CTC counts will be identified at baseline, 1-2 weeks after the therapy, and at each restaging, for up to 1 year.	200 MBC patients	Molecular and genetic profiling toward personalized treatment recommendation	Recruiting
**NCT05360290**	An interventional, multicenter prospective study	To evaluate the prognostic value of CTCs in BC patients receiving neoadjuvant therapy and surgery. Correlations with iDFS, OS, and pCR will be analyzed.	484 BC patients (a metastatic stage IV excluded)	Device GILUPI CellColector®	Not yet recruiting
**NCT04993014**	An interventional, unicentric randomized phase II study	To compare DFS between HER-2 positive patients with detected CTCs vs. negative/absent CTCs at baseline and assess the relationship between HER2 positivity and CTC counts/ml. Moreover, the results of CTC detection and ER/HER2 status of CTCs at baseline will be correlated with all molecular, imaging, and clinical follow-up data in a 3-5 year period.	80 HER2-positive BC (stage I to III)	Drugs: Pertuzumab, Trastuzumab HER2 positive CTCs patients, randomized for adjuvant trastuzumab versus trastuzumab + pertuzumab HER2 absent CTCs patients, randomized for adjuvant trastuzumab versus trastuzumab + pertuzumab	Recruiting
**NCT01957332**	An interventional open-label study	To evaluate the clinical utility of experimental PET scans. A correlation of CTC analysis, as well as CTC count and ER/HER2 status of CTCs at baseline, will be performed with all molecular, imaging, and clinical follow-up data in a 3-5 year period.	217 MBC patients	Molecular imaging Non-invasive 18F-fluoroestradiol (18F-FES)-PET and Zirconium-89 (89Zr)-trastuzumab-PET scan techniques	Active, not yet recruiting
**NCT05326295**	An observational retrospective study	To determine the impact of CTCs surveillance on predicting the response to neoadjuvant chemotherapy, surgery, and adjuvant chemotherapy. The efficacy of CTCs surveillance in predicting iDFS, OS, and metastasis will be evaluated, together with the expression of PDL1 and FOXC3 on CTCs will be evaluated.	1000 early-stage BC	CTCs separation and enrichment technology.	Recruiting

**NCT03213041**	An interventional, a single-arm open-label phase II study	To evaluate the impact of carboplatin - pembrolizumab treatment on PFS and OS in CTC-positive patients. In addition, ORR and CBR will be assessed. PDL-1 expression in CTCs will be correlated with therapeutic benefits.	100 MBC	Drug: Carboplatin<br>Biological: Pembrolizumab	Recruiting
**NCT04504747**	An observational study	To study drug resistance in BC patients receiving neoadjuvant chemotherapy via molecular analyses of patients′ biopsies, patient-derived organoids, and CTCs isolated before/during/after neoadjuvant treatment.	150 non-metastatic BC	Molecular analysis of blood and tumor samples	Not yet recruiting
**NCT03872388**	An interventional, non-randomized open-label phase II study	To identify the patients with undetectable CTCs at six months who did not achieve a pCR or RCB-I after receiving neoadjuvant chemotherapy with and without atorvastatin therapy. The study will investigate whether the baseline level of CTCs and CTC changes are associated with a 2-year RFS rate.	80 TNBC patients stages IIb-III	Drugs: Atorvastatin, Capecitabine	Recruiting
**NCT00877500**	An interventional, randomized open-label phase II study	To verify the genomic and proteomic characteristics associated with the disability to achieve a pCR after neoadjuvant systemic therapy and correlate these features with patient outcomes. The presence of CTCs will be validated at baseline, during and after ixabepilone therapy, or during observation.	116 HER2/Neu negative invasive BC	Drug: Ixabepilone	Active, not yet recruiting
**NCT01785420**	An interventional, randomized, double-masking open-label phase III study	To uncover the effect of the short duration of peri-operative Trastuzumab on DFS in operable BC. CTCs counts will be assessed in the peripheral blood prior to pre-operative therapy, 10 minutes prior to the surgery, during surgery, and ten days after surgery on 40 consecutive patients (20/20 in experimental and control arms, respectively).	1100 BC patients HER2-positive (stage I)	Drug: Trastuzumab	Recruiting
**NCT04158362**	An interventional, randomized, open-label phase III study	To compare the efficacy of standard endocrine therapy + abemaciclib vs. standard chemotherapy and evaluate the differences in PFS within 24 weeks. The study also aims to determine the predictive and prognostic value of CTC count (<5 versus ≥ 5 CTCs/7.5mL).	378 MBC patients ER+/HER2-negative	Drugs: Paclitaxel injection, Capecitabine tablets, Letrozole 2.5mg, Anastrozole 1mg, Fulvestrant Prefilled Syringe, Abemaciclib	Recruiting

**NCT02344472**	An interventional, randomized, open-label phase III Study	To measure the CTC counts in the peripheral blood at the baseline and at different time points of palliative care, and to assess the number of CTCs for treatment response prediction.	217 MBC patients HER2/HR-positive	Drugs: Pertuzumab, Trastuzumab, Doxetacel, Paclitaxel, Vinorelbine, Ribociclib, Fulvestrant, nab-Paclitaxel, Anastrozole, Letrozole, Fulvestrant, Eribulin, Leuprorelin, Goserelin, Exemestane, Capecitabine	Recruiting
**NCT01706432**	An observational, prospective case study	To study the changes in the number of CTCs at baseline, 3-4 weeks post-treatment and each 9-12 weeks in a one-year period in patients receiving hypofractionated image-guided radiation therapy.	4 MBC patients	Hyperfractionated radiation therapy stereotactic radiosurgery	Active, not yet recruiting
**NCT00773695**	An interventional, a multicenter, randomized phase III Study	To assess the percentage of patients with treatment-induced changes in the number of CTCs in peripheral blood at baseline up to the end of study treatment (approximately 24 weeks).	150 primary BC patients HER-negative (a metastatic stage excluded)	Drugs: Aromatase Inhibitor, Bevacizumab, Epirubicine, 5-Fluorouracil (5FU), Cyclophosphamide, Paclitaxel, Docetaxel	Active, not yet recruiting
**NCT03473639**	An interventional, single-arm open-label study	To detect CTC levels in peripheral blood of high-risk BC after neoadjuvant therapy before and at the end of the treatment. Subsequently, the study aims to evaluate the relationship between CTCs and residual disease at the surgery and DFS.	55 MBC and high-risk BC patients after neoadjuvant therapy	Drugs: Entinostat, Capecitabine	Recruiting
**NCT03818685**	An interventional, a multicenter, randomized open-label phase II study	To monitor circulating immune and tumor cells in the blood of TNBC patients with residual disease after neoadjuvant chemotherapy, treated with post-operative adjuvant therapy combining radiotherapy at specific time points (cycle 1, 2, 5, and 24 months after randomization) or in case of relapse. Each cycle represents 21 days.	95 TNBC patients	Drugs: Nivolumab, Ipilimumab, Capecitabine Radiotherapy	Active, not yet recruiting
**NCT04703244**	An observational, case-only prospective study	To prepare patient-derived xenografts and organoids from BC patients after chemotherapy or endocrine therapy. The number of CTCs will be detected in blood samples and further correlated with the residual cancer burden.	100 BC patients with residual disease after neoadjuvant chemotherapy	Chemotherapy or endocrine therapy for breast cancer	Recruiting

**NCT04526587**	An observational, prospective study	To isolate CTC from blood samples of CDK4/6 inhibitor-treated patients at specified time points for the development of 3-dimensional organoid cultures and CTC-derived xenografts.	300 MBC patients HER2-negative/ ER+	Cyclin-dependent kinase 4/6 (CDK4/6) inhibitors cciclib-based therapies	Recruiting
**NCT04646564**	An interventional, randomized, open-label, phase III Study	To evaluate CTCs as prognostic and predictive markers of survival, and early detection of progression in patients with extracranial oligometastatic BC disease.	170 MBC patients	Standard systemic therapy, including chemotherapy, endocrine therapy, targeted therapy, or immunotherapy Radiotherapy	Recruiting

Abbreviations: BC, breast cancer; CBR, clinical benefit rate; CTC, circulating tumor cell; DFS, disease-free survival; ER, estrogen receptor; iDFS, invasive disease-free survival; MBC, metastatic breast cancer; ORR, objective response rate; OS, overall survival; pCR, pathologic complete response; PET, positron emission tomography; PFS, progression-free survival; RCB-I, residual cancer burden-I; RFS, recurrence-free survival; TNBC, triple-negative breast cancer
